# Bioactive Components of Chinese Propolis Water Extract on Antitumor Activity and Quality Control

**DOI:** 10.1155/2016/9641965

**Published:** 2016-03-30

**Authors:** Hongzhuan Xuan, Yuehua Wang, Aifeng Li, Chongluo Fu, Yuanjun Wang, Wenjun Peng

**Affiliations:** ^1^Key Laboratory of Pollinating Insect Biology, Ministry of Agriculture, Beijing 100093, China; ^2^School of Life Science, Liaocheng University, Liaocheng 252059, China; ^3^College of Chemistry and Chemical Engineering, Liaocheng University, Liaocheng 252059, China

## Abstract

To understand the material basis of antitumor activity of Chinese propolis water extract (CPWE), we developed a simple and efficient method using macroporous absorptive resin coupled with preparative high performance liquid chromatography and separated and purified eleven chemical components (caffeic acid, ferulic acid, isoferulic acid, 3,4-dimethoxycinnamic acid, pinobanksin, caffeic acid benzyl ester, caffeic acid phenethyl ester, apigenin, pinocembrin, chrysin, and galangin) from CPWE; then we tested the antitumor activities of these eleven components using different human tumor cell lines (MCF-7, MDA-MB-231, HeLa, and A549). Furthermore, cell migration, procaspase 3 level, and reactive oxygen species (ROS) of effective components from CPWE were investigated. Our data showed that antitumor activities of the eleven components from CPWE were different from each other. CPWE and its effective components induced apoptosis by inhibiting tumor cell migration, activating caspase 3, and promoting ROS production. It can be deduced that the antitumor effects of propolis did not depend on a single component, and there must exist “bioactive components,” which also provides a new idea for Chinese propolis quality control.

## 1. Introduction

Propolis is a resinous substance collected by* Apis mellifera* from various tree buds, and it has been used as a folk medicine since ancient time for its widely biological properties, such as antibacterial, antiviral, antioxidant, anti-inflammatory, immunomodulatory, and antitumor. [1–5]. However, in propolis application the biggest problem is the instability of its therapeutic effects and the material basis has not been fully understood, which is caused by the imperfection of propolis quality control and evaluation system. Propolis quality control system is difficult to be established, for there are more than 600 constituents identified from different kinds of propolis in the world, such as polyphenols (flavonoids, phenolic acids, and their esters), terpenoids, steroids, and amino acids [6–8]. And the other major cause is that there is not a unified extract method and solvent process. Ethanol is the most common solvent during propolis extracting process, and most of the studies and biological activities in propolis are based on propolis ethanolic extract (PEE), which leads to little knowledge known about the biological activities of the propolis water extract, especially “poplar propolis” from China [9, 10].

Recently, we developed a simple and efficient method using macroporous absorptive resin (MAR) coupled with preparative high performance liquid chromatography (PHPLC) for separation of polyphenols from Chinese propolis water extract (CPWE). Six phenolic acids and five flavonoids (caffeic acid, ferulic acid, isoferulic acid, 3,4-dimethoxycinnamic acid, pinobanksin, caffeic acid benzyl ester, caffeic acid phenethyl ester (CAPE), apigenin, pinocembrin, chrysin, and galangin) with high purities were isolated, and the chemical structures were further confirmed by UV and NMR analysis [11].

Considering the imperfection of Chinese propolis quality control system and the ambiguity of material basis of antitumor activity of CPWE, in present study we studied the antitumor activities of CPWE and the eleven isolated components from CPWE to determine bioactive components of antitumor activity and provide a new idea for Chinese propolis quality control.

## 2. Materials and Methods

### 2.1. Chemicals and Reagents

Dulbecco's modified Eagle's medium (DMEM) was from Gibco (USA). Fetal bovine serum (FBS) was from Hyclone Lab Inc. (USA). Sulforhodamine B (SRB), Hoechst 33258, and 2′,7′-dichlorodihydrofluorescein (DCHF) were from Sigma Co. (USA). Acridine orange was from Amresco (USA). Primary antibodies against *β*-actin and secondary antibody (horseradish peroxidase) were from Santa Cruz Biotechnology (USA). Primary antibody against procaspase 3 was from Cell Signaling Technology (USA). All other reagents were ultrapure grade.

### 2.2. Preparation of Propolis Extracts

Chinese propolis was obtained from colonies of honeybees,* A*.* mellifera* L., in Shandong province of north China and the main plant origin was poplar (*Populus* sp.). Chinese propolis 0.25 kg was frozen, milled, and extracted with boiling water. The water extract was filtered, combined, and concentrated under reduced pressure with a rotary evaporator. Then 95% ethanol was added to the solution to remove polysaccharide until the concentration of ethanol was about 70%. After 12 h, the supernatant was separated and concentrated under reduced pressure. The water-soluble fraction was first “prefractioned” by MAR to obtain four subfractions; and they were all subjected to PHPLC to get different components [11].

### 2.3. Cell Culture

The human breast cancer cells, MCF-7 (human breast cancer ER (+)) and MDA-MB-231 (human breast cancer ER (−)) cells, lung cancer A549 cells, and human colonic carcinoma HeLa cells were purchased from American Type Culture Collection (ATCC, USA). MCF-7, MDA-MB-231, A549, and HeLa cells were cultured in DMEM medium supplemented with heat-inactivated 10% FBS and 100 U/mL of penicillin and 100 *μ*g/mL streptomycin. Cells were incubated at 37°C in a humidified atmosphere of 5% CO_2_ and 95% air.

### 2.4. Cell Viability Assay

Four different tumor cells were seeded onto 96-well plates and treated with different components separated from CPWE (20, 40, 80, and 160 *μ*M) for 24 and 48 h, respectively. Cell viability was determined by SRB assay. In detail, fix cells by adding 100 *μ*L of cold 10% trichloroacetic acid and incubate for 1 h at 4°C, and then wash the plates with deionized water five times. Add 50 *μ*L of 0.4% SRB solution to each well and shake for 5 min on titer plate shaker. Wash the plate with 1% acetate five times, and subsequently add 100 *μ*L of 10 mM Tris base to dissolve the bound dye. Mix for 5 min on a microtiter plate shaker and read optical densities at the wavelength of 492 nm using Multiskan MK3 microplate reader (Thermo Co., USA). The viability (%) was expressed as (OD of treated group/OD of control group) × 100%. The viability of the control cells was set to 100%.

### 2.5. Nuclear Fragmentation Assay

The morphological changes of nuclei of MCF-7 cells treated with different components from CPWE were detected by acridine orange staining. At 48 h, cells were washed gently with 1x PBS once and then stained with 5 *μ*g/mL acridine orange at room temperature for 1 min, after that they were washed gently twice to be observed under a TE2000S fluorescence microscope (Nikon, Japan).

### 2.6. Hoechst 33258 Staining

Hoechst 33258 staining was used to observe apoptotic morphology of MCF-7 cells treated with different components from CPWE. At 48 h, cells in all groups were stained with 10 *μ*g/mL Hoechst 33258 for 15 min and then were gently washed with 1x PBS once. Nuclear condensation and fragmentation were observed under a TE2000S fluorescence microscope (Nikon, Japan).

### 2.7. Wound-Healing Assay

MDA-MB-231 cells were grown to 80% confluence in a 24-well plate; then the monolayers were scratched with a plastic tip, washed by 1x PBS to remove floating cell debris, and then incubated in medium in the absence or presence of different components from CPWE for 48 h. Cell migration into the wound surface was determined under a TE2000S inverted microscope (Nikon, Japan). Migrated cells across the scratched lines were counted by Image-Pro Plus software (USA).

### 2.8. Western Blotting Analysis

Western blotting analysis was used to determine the protein levels in cells treated with different components from CPWE. Cells were collected and lysed in the lysis buffer, and protein concentration was measured by Bradford method as previously described [12]. Protein (30 *μ*g) was separated by running through 12% SDS-PAGE gel and transferred to the PVDF membrane. The transferred proteins were visualized with an enhanced chemiluminescence detection kit.

### 2.9. Measurement of ROS Production

ROS production in MCF-7 cells treated with different components from CPWE was determined by use of a fluorescent probe, DCHF as previously described [13]. The fluorescence was observed on a laser scanning confocal microscopy (Olympus FV1200, Japan). ROS level was quantified by Image-Pro Plus software (USA). Results were shown as relative fluorescence intensity of three independent experiments.

### 2.10. Statistical Analysis

All experiments were performed in duplicate and repeated at least 3 times. Data are expressed as means ± SEM. Statistical analyses were performed using independent *t*-tests and analysis of variance (ANOVA) followed by the Tukey* post hoc test*. A *P* < 0.05 was considered significant.

## 3. Results

### 3.1. Major Components of CPWE

**Figure 1:**
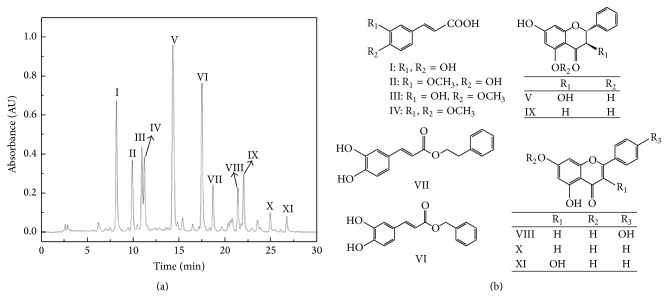
HPLC chromatograms of the crude Chinese propolis water extract (CPWE) and the chemical structure of the eleven components. I: caffeic acid, II: ferulic acid, III: isoferulic acid, IV: 3,4-dimethoxycinnamic acid, V: pinobanksin, VI: caffeic acid benzyl ester, VII: caffeic acid phenethyl ester, VIII: apigenin, IX: pinocembrin, X: chrysin, and XI: galangin.

Finally, eleven components from CPWE were obtained including I: caffeic acid (30 mg), II: ferulic acid (16 mg), III: isoferulic acid (10 mg), IV: 3,4-dimethoxycinnamic acid (12 mg), V: pinobanksin (42 mg), VI: caffeic acid benzyl ester (36 mg), VII: caffeic acid phenethyl ester (12 mg), VIII: apigenin (8 mg), IX: pinocembrin (11 mg), X: chrysin (5 mg), and XI: galangin (4 mg). Their purities were all above 98% as determined by HPLC, and the chemical structures (shown in Figure 1) were confirmed by UV and NMR analysis.

### 3.2. Effects of the Eleven Components Isolated from CPWE on the Proliferation of Four Tumor Cell Lines

**Figure 2:**
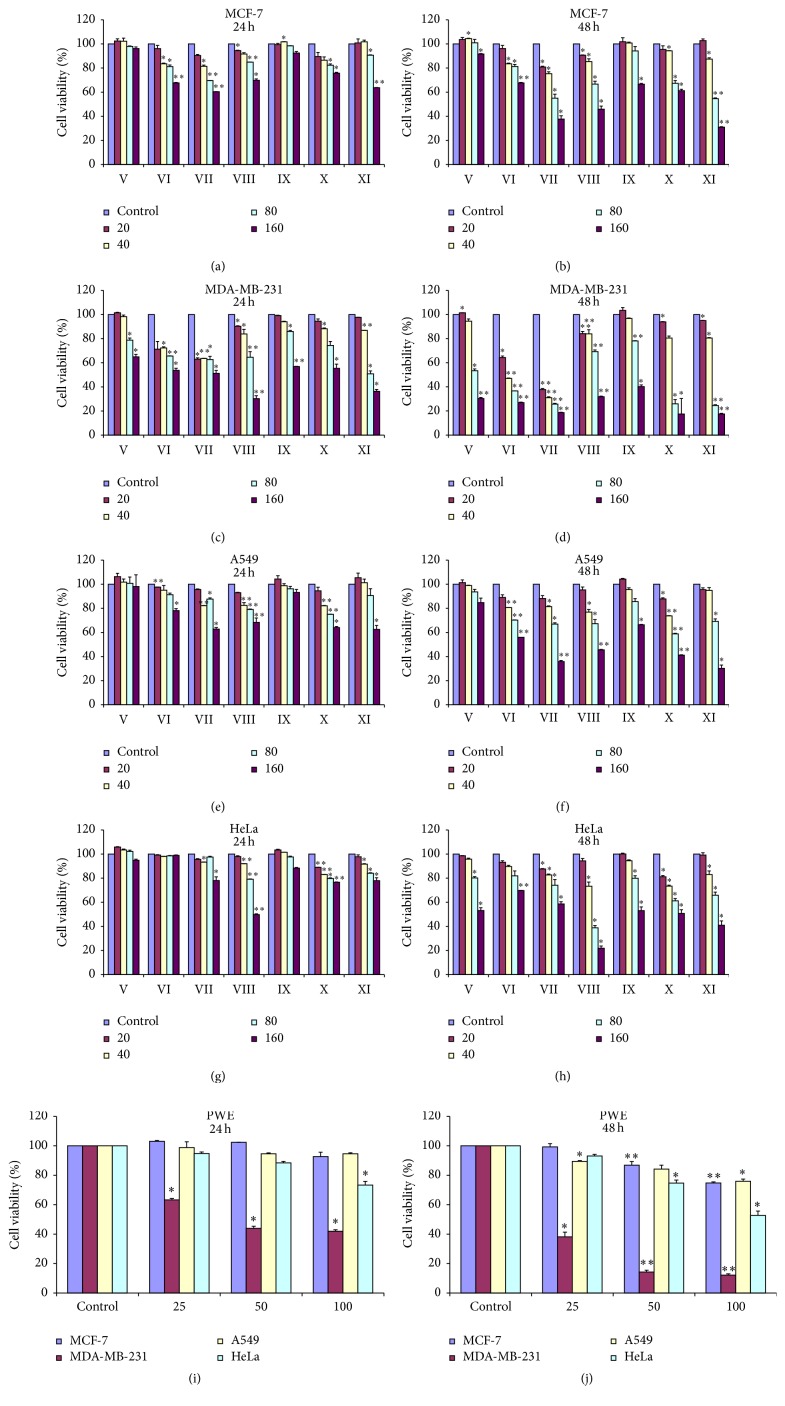
Effects of the seven components isolated from CPWE on the proliferation of four tumor cells. (a) and (b), effect of the seven components (20, 40, 80, and 160 *μ*M) on MCF-7 cell viability at 24 and 48 h. (c) and (d), effect of the seven components on MDA-MB-231 cell viability at 24 and 48 h. (e) and (f), effect of the seven components on A549 cell viability at 24 and 48 h. (g) and (h), effect of the seven components on viability of HeLa cell viability at 24 and 48 h. (i) and (j), effect of crude CPWE on proliferation of four tumor cells. Cell viability was tested by SRB assay and illustrated in column figures (^*∗*^ *P* < 0.05, ^*∗∗*^ *P* < 0.01 versus control, *n* = 3). Data are means ± SEM.

We investigated the sensitivity of four tumor cell lines to the eleven components (20, 40, 80, and 160 *μ*M) and CPWE (25, 50, and 100 *μ*g/mL) for 24 and 48 h using SRB assay at 24 and 48 h. Caffeic acid, ferulic acid, isoferulic acid, and 3,4-dimethoxycinnamic acid had no significant cytotoxicity to four tumor cells (data were not shown); the other seven components significantly inhibited four tumor cells' proliferation in a dose- and time-dependent manner. The crude CPWE also inhibited cell proliferation of four tumor cells; however, the inhibitory effect of CPWE was lower than that of ethanol-extracted Chinese propolis, which was tested previously [14]. Furthermore, the sensitivity of four tumor cell lines to the seven components and CPWE from strong to weak was followed by MDA-MB-231, HeLa, A549, and MCF-7 cells (^*∗*^ *P* < 0.05, ^*∗∗*^ *P* < 0.01; Figure 2).

Notably, the cytotoxicity of pinocembrin to tumor cells was higher than pinobanksin; CAPE was higher than caffeic acid benzyl ester, and the most effective antitumor concentration for seven different components was at concentration higher than 80 *μ*M, so we used 80 *μ*M for the seven components and CPWE 100 *μ*g/mL as the following study dose.

### 3.3. Effects of the Seven Different Components and CPWE on Apoptosis in MCF-7 Cells

**Figure 3:**
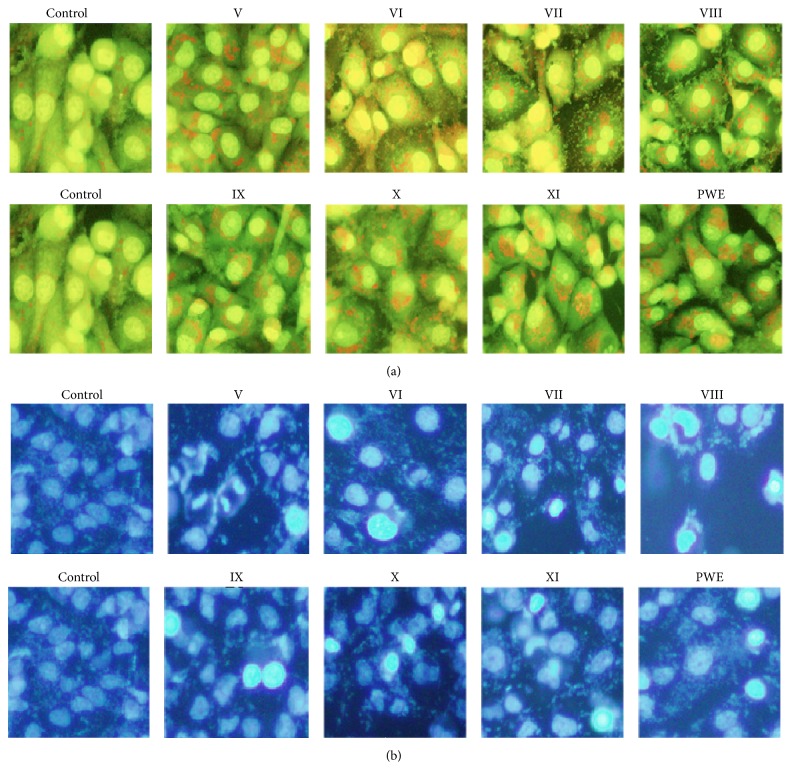
Effects of the seven components and crude CPWE on nuclear fragmentations of MCF-7 cells. (a) Morphological changes of nuclei by staining with acridine orange at 48 h (×200). (b) Morphological changes of nuclei by staining with Hoechst 33258 at 48 h (×200).

Acridine orange staining and Hoechst 33258 staining results indicated that the seven different components at concentration of 80 *μ*M and CPWE (100 *μ*g/mL) evidently induced nuclear condensation and fragmentation in MCF-7 cells (Figure 3).

### 3.4. Effects of the Seven Different Components and CPWE on MDA-MB-231 Cells Migration

**Figure 4:**
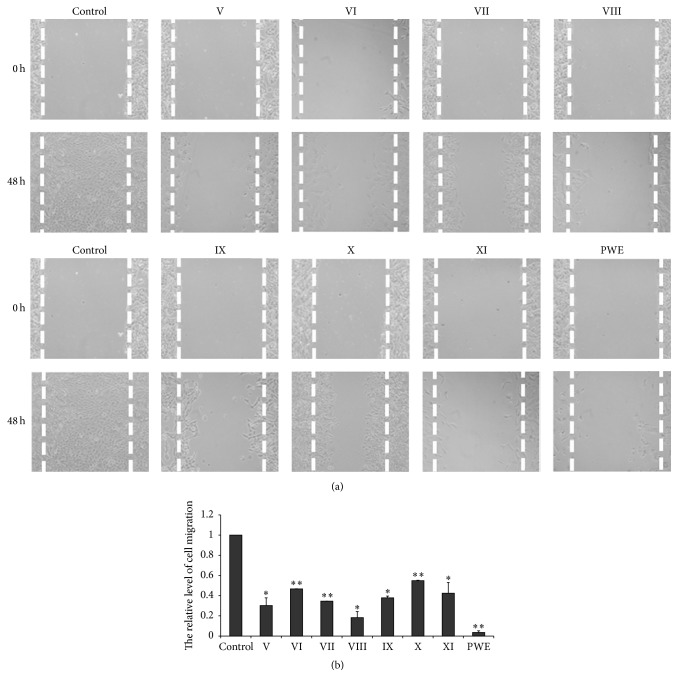
Effects of the seven components and crude CPWE on migration of MDA-MB-231 cells. (a) Cell migration micrographs obtained under a phase contrast microscope at 0 and 48 h (×100). (b) Relative levels of cell migration (^*∗*^ *P* < 0.05, ^*∗∗*^ *P* < 0.01 versus control, *n* = 3).

The migrations of MDA-MB-231 cells were detected by wound-healing assay after being treated with the seven different components at concentration of 80 *μ*M and CPWE (100 *μ*g/mL); the results indicated that the seven different components and CPWE significantly inhibited MDA-MB-231 cells migration at 48 h (Figure 4).

### 3.5. Effects of the Seven Different Components and CPWE on the Level of Procaspase 3 in Two Breast Cancer Cells

**Figure 5:**
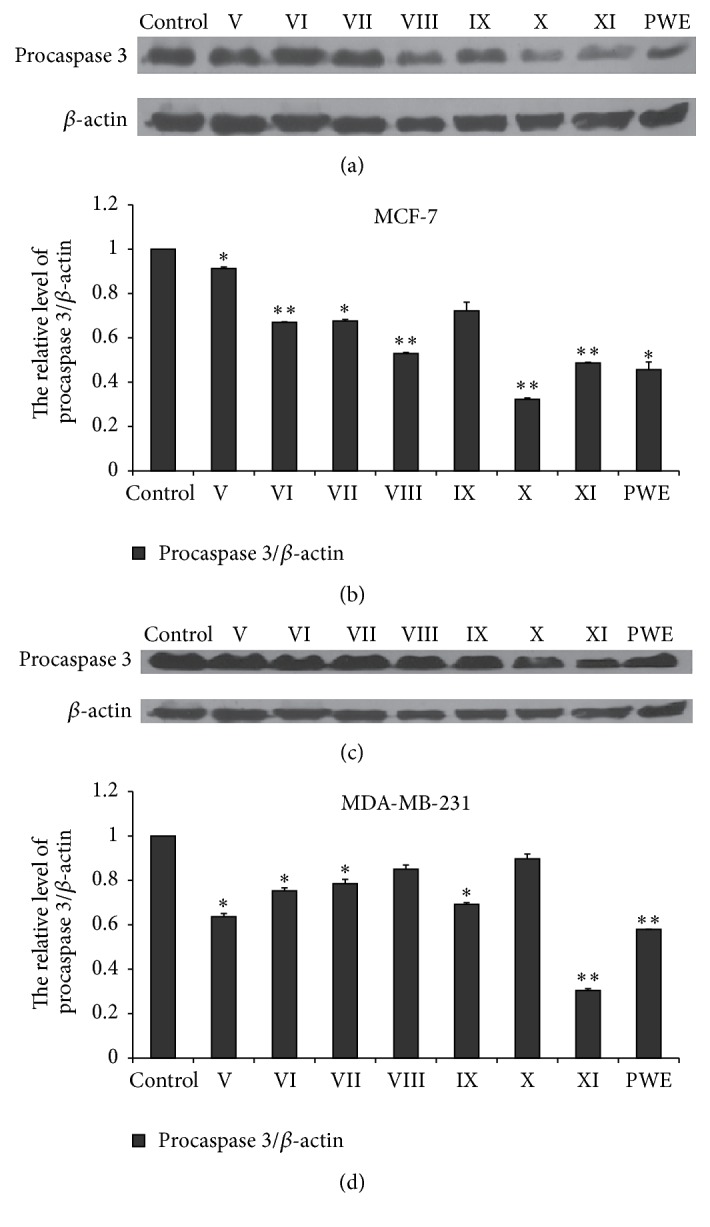
Effects of the seven components and crude CPWE on the expression of procaspase 3 in MCF-7 and MDA-MB-231 cells. (a) and (c), expression of procaspase 3 in MCF-7 and MDA-MB-231 cells at 24 h, respectively; (b) and (d) quantification of relative expression quantity in MCF-7 and MDA-MB-231 cells at 24 h, respectively (^*∗*^ *P* < 0.05, ^*∗∗*^ *P* < 0.01 versus control, *n* = 3).

The seven different components at concentration of 80 *μ*M and CPWE (100 *μ*g/mL) significantly activated caspase 3 by western blotting assay in MCF-7 and MDA-MB-231 cells (Figure 5).

### 3.6. Effects of the Seven Different Components and CPWE on the Production of ROS in MCF-7 Cells

**Figure 6:**
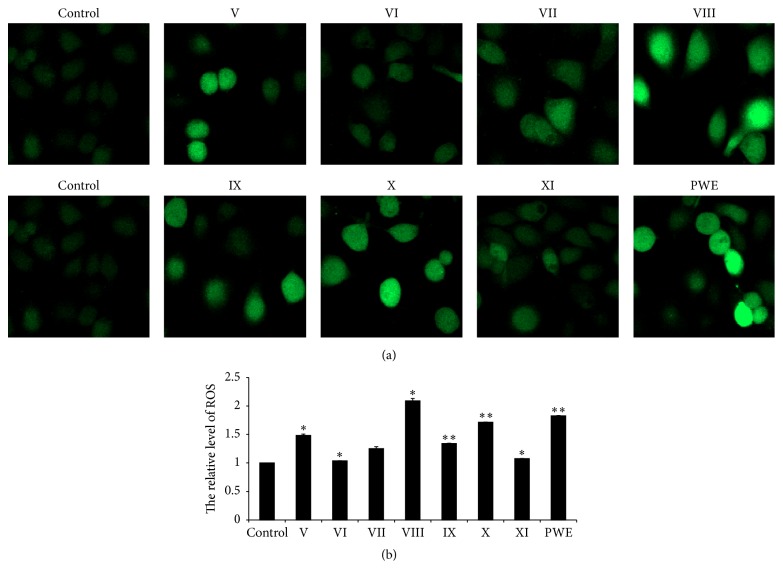
Effects of the seven components and crude CPWE on the production of reactive oxygen species (ROS) in MCF-7 cells. (a) Fluorescent micrographs obtained at 48 h. (b) Quantification of relative quantity of ROS in MCF-7 cells. Values represent the relative fluorescent intensity per cell determined by laser scanning confocal microscopy (^*∗*^ *P* < 0.05, ^*∗∗*^ *P* < 0.01 versus control, *n* = 3).

The seven different components at concentration of 80 *μ*M and CPWE (100 *μ*g/mL) obviously affected ROS production in MCF-7 cells although the ROS levels were different from each other (Figure 6).

## 4. Discussion

Previous studies from our group reported the biological activities of Chinese propolis [15–17] and the present study was the first one to investigate the effective components on antitumor activity in CPWE. Four phenolic acids (caffeic acid, ferulic acid, isoferulic acid, and 3,4-dimethoxycinnamic acid) had little cytotoxicity on four tumor cell lines; the other seven constituents (pinobanksin, caffeic acid benzyl ester, caffeic acid phenethyl ester, apigenin, pinocembrin, chrysin, and galangin) obviously decreased four tumor cells' proliferation, although the inhibitory effects of the seven components were different from each other, which indicated that the antitumor effects of CPWE did not depend on a single component, and at least the seven effective components might be “bioactive components” of antitumor activity. Admittedly, there must be other effective components needed to be studied further.

The standardization of Chinese propolis has caused some interest in recent years in China, and HPLC fingerprint of Chinese propolis from different regions, sources, and seasons has been fully studied, and the authentication standard of Chinese propolis and poplar buds had also been established [18, 19], which greatly promoted the research of quality control system of Chinese propolis. It was pointed out that chrysin, catechol, or another component from propolis could be a candidate for the standardization of Chinese propolis [20, 21]. However, there still exist a lot of problems. For example, propolis has similar biological activities although chemical components vary greatly [22]. And more importantly, a number of studies have confirmed that propolis and its plant sources, poplar buds or gums, have similar biological activities. Wang et al. indicated that ethanol extracts of Chinese propolis (EECP) and buds from poplar had similar anti-inflammatory effects* in vivo* and* in vitro* [23]. Another report suggested that the antioxidant mechanisms of EECP and poplar gums were similar, but they also indicated that the antioxidant activities of EECP were stronger than poplar gums. Further analysis indicated that the total content of eight components from EECP (caffeic acid, ferulic acid,* p*-coumaric acid, apigenin, chrysin, pinocembrin, CAPE, and galangin) was 5.85 g/100 g. However, in poplar gums, caffeic acid, ferulic acid, and* p*-coumaric acid were not identified, and the total content of the other five components was only 2.59 g/100 g [24]. Based on the facts we deduced that the major cause for EECP with a higher antioxidant than poplar gums was that EECP had more effective components. In present study, we further confirmed that it was not a single component playing the antitumor activity in propolis. Thus, the quality evaluation system of Chinese propolis might be imperfect if it is only based on the quantitative analysis of chemical composition of propolis or some single component, and here we proposed that it was acceptable to perfect the quality evaluation system of Chinese propolis based on “bioactive components.”

The major mechanism of inhibiting tumor cell proliferation of the seven effective components from CPWE was to induce apoptosis by activating caspase 3, the executor of apoptosis, and induce ROS production, which was consistent with our previous studies [14].

In summary, our data highlight the effective components of CPWE on antitumor activity and the probable action mechanisms in inhibiting tumor cell proliferation and provide a novel idea for Chinese propolis quality control.
